# Recidivism and Rehabilitation of Criminal Offenders: A Carrot and Stick Evolutionary Game

**DOI:** 10.1371/journal.pone.0085531

**Published:** 2014-01-16

**Authors:** Bijan Berenji, Tom Chou, Maria R. D'Orsogna

**Affiliations:** 1 Department of Biomathematics, University of California Los Angeles, Los Angeles, California, United States of America; 2 Department of Mathematics, California State University at Northridge, Los Angeles, California, United States of America; University of Maribor, Slovenia

## Abstract

Motivated by recent efforts by the criminal justice system to treat and rehabilitate nonviolent offenders rather than focusing solely on their punishment, we introduce an evolutionary game theoretic model to study the effects of “carrot and stick” intervention programs on criminal recidivism. We use stochastic simulations to study the evolution of a population where individuals may commit crimes depending on their past history, surrounding environment and, in the case of recidivists, on any counseling, educational or training programs available to them after being punished for their previous crimes. These sociological factors are embodied by effective parameters that determine the decision making probabilities. Players may decide to permanently reform or continue engaging in criminal activity, eventually reaching a state where they are considered incorrigible. Depending on parameter choices, the outcome of the game is a society with a majority of virtuous, rehabilitated citizens or incorrigibles. Since total resources may be limited, we constrain the combined punishment and rehabilitation costs per crime to be fixed, so that increasing one effort will necessarily decrease the other. We find that the most successful strategy in reducing crime is to optimally allocate resources so that after being punished, criminals experience impactful intervention programs, especially during the first stages of their return to society. Excessively harsh or lenient punishments are less effective. We also develop a system of coupled ordinary differential equations with memory effects to give a qualitative description of our simulated societal dynamics. We discuss our findings and sociological implications.

## Introduction

The emergence of human cooperation is a subject of great interest within the behavioral sciences. In recent years several studies have tried to understand why such an exceptional level of cooperation among humans exists despite individual gains that may be attained if people acted selfishly. Some of the current hypotheses to explain large scale cooperation are based on player reciprocity, status, or altruistic and tit–for–tat behaviors between two actors [1]–[4]. One of the most endorsed theories however includes third party punishment, where defectors are punished for following their self–serving interests [5], [6].

Game theory has often been used as a tool to explore human or animal behavior since its mathematical framework allows for the study of the dynamics of players and their choices in a systematic, albeit simplified, way. As a result, many authors within several disciplines have developed and analyzed games that include the effects of punishment as a way to foster cooperation among humans [7]–[9]. Most, but not all, of these studies are based on the classic prisoner's dilemma paradigm [10] and include elements such as the severity of sanctions and the willingness of participants to punish offenders [11], the frequency and expectation of enforcement [12], collective punishment and rewards [13]–[15], network structures [16] and the possibility of directly harming adversaries[17]–[19]. On the other hand, very little work has focused on studying recidivism by offenders after punishment and how prevention measures – and not only punishment – taken by third parties may improve recidivism rates and affect cooperation.

In this paper we focus on recidivism and rehabilitation within the specific context of criminal behavior, where cooperators are law abiding citizens and where defectors are criminals that may be punished if apprehended. We introduce a dynamic game-theoretic model to study how player choices change over time not only due to punishment after an offense, but also due to possible post–punishment intervention given by third parties as prevention against future crimes, in the form of housing, job, training or family assistance. In our “carrot vs. stick” game we start from non–offenders who are progressively exposed to opportunities to commit crimes. The probability of offending is dependent on external factors, such as societal pressure or the threat of punishment, and internal ones, such as the player's criminal history. Since we assume that repeat offenders are provided with assistance upon release, the probability to commit a crime also depends on the quality and duration of any previously assigned post–release assistance. Finally, to model the limited resources available to law enforcement agencies [20], [21], we assume that the combination of punishment and post–release program costs per incarceration are fixed: the more punishment a player is subject to, the less post–release intervention assistance he or she will receive.

The rules of our game are chosen so that players will progress in their criminal careers as recidivists, until they are considered incorrigible, or choose to shun their criminal lives and become virtuous citizens. In this way, an initial society will evolve towards a final configuration comprised solely of incorrigibles or virtuous citizens. From a mathematical standpoint our evolutionary game will include history dependent strategies so that individuals placed in the same circumstances may choose different courses of action depending on their past crimes. Furthermore since each player's choices depend on the entire societal makeup, our model includes global interactions.

We will analyze the ratio of the two final populations as a function of relevant parameters and show that under certain circumstances, post–release intervention programs, if structured to be long lasting, may have important consequences on the final societal makeup and be more effective than punishment alone. In particular, we will show that the ratio of incorrigibles to virtuous citizens may be optimized by properly balancing available resources between punishment and post–release assistance. Indeed, this is the main result of our paper: that punishment and assistance are effective, complementary tools in reducing crime, and that a judicious application of both will yield better results than focusing solely on either one.

It is important to note that while several “carrot and stick” evolutionary games have been introduced in the context of public goods games [14], [22], [23], in most cases, the carrot and the stick are mutually exclusive. Players are either rewarded for their cooperative actions or punished for their selfish behavior, but not subject to both incentives and punishment at the same time. In our work instead, all criminal-defectors are subject first to the stick, via the punishment phase, and later to the carrot, in the rehabilitation phase. As mentioned above, if the total amount of resources to be spent on each criminal is finite, then the optimal way of reducing crime a balanced approach, where criminals are punished adequately while at the same time receiving enough incentives for rehabilitation.

In the remainder of this Introduction, we motivate our work by including a brief discussion on recidivism and rehabilitation. In the Analysis section we introduce our dynamical game and justify the variable and parameter choices made to model societal trends. We present our numerical findings in the Results section where we also derive a set of coupled ordinary differential equations with memory to describe the dynamics more succinctly. We show that the two approaches – simulations and solving coupled ordinary differential equations – lead to qualitatively similar results. We end with a brief Summary and Discussion where we discuss findings from our “carrot and stick” game and their sociological implications.

### Sociological background

Starting from the 1970s, the severity of punishment for criminal offenses in the United States has been steadily increasing, as evidenced by growing incarceration rates, swelling prison populations, longer sentencing and the increasing popularity of mandatory minimum sentencing policies, such as “three strikes” laws[24], [25]. At present, the United States has one of the highest incarceration rates in the western world, with about one percent of the population imprisoned at any given time [26]. The cost incurred by the taxpayer to fund the criminal justice system – including day to day expenditures, facility maintenance and construction, court proceedings, health care and welfare programs – is estimated to be a staggering 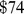 billion for 2007 alone[27]. Related social problems include prison overcrowding and violence, racial inequities, broken families left behind, and releasing into the community individuals who have not been rehabilitated during their prison time and are ill–equipped to lead a crime free life after being released to the larger society.

One of the prevailing schools of thought is that the severity, unpleasantness and social stigma of life in prison may serve as deterrents to future criminal behavior, promoting the principle that “crime does not pay”[28]. Opposing points of view contend that due to the mostly poor conditions within prisons and lack of opportunities for change, most inmates will be returned to society hardened and, having been exposed to an environment dominated by more experienced criminals, more savvy and likely to offend again. Indeed, several criminological studies have shown that harsher sentences do not necessarily act as deterrents and may even slightly increase the likelihood of offending [29]–[31]. On the other hand, social intervention and support combined with punishment and coercion have been shown to be effective in preventing crimes[32], [33].

Recidivism rates in the United States vary depending on crime. In the case of property and drug related offenses, the likelihood of rearrest within three years after release is about 70 percent[29], higher than that of most western countries. In recent years thus, due to mounting incarceration costs and high recidivism rates, law enforcement and correction agencies have begun turning to novel approaches, designed to offer rehabilitation programs to prisoners during incarceration and assistance upon release. Such programs include counseling to increase self-restraint, drug treatment, vocational training, educational services, housing and job assistance, community support, helping rekindle family ties, and even horticulture[34], [35]. The issue is a multifaceted one and for former inmates, the question of whether or not to re-offend is a highly individual one that depends on their personal histories[29], [36], their experiences while in jail, and the environment they are released to[29]. In general, the most successful intervention programs have been the ones that offered the most post–release assistance[37].

### Analysis

In this section we present the evolutionary game theory model we developed as inspired by the sociological observations described above. We consider a population of 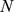 individuals where each player carries his or her specific history of 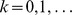 past offenses, whether punished or unpunished. Thus, at any time we have sub-populations of 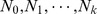 individuals with a record of past 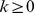 crimes.

We assume that when faced with the opportunity to commit a crime, players may decide to offend and transition from state 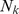 to 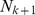. If they choose not to commit a crime, they may either remain in state 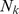 or choose to shun criminal activity altogether, for any and all future opportunities. Individuals who decide never to commit crimes again in the future, regardless of record and circumstances, are called paladins. Since paladin behavior is fixed, we take these individuals out of the game as active players and place them in the subpopulation 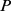. Note that the difference between paladins 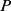 and players in the 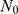 subpopulation is that a paladin may have committed crimes in the past, but will not commit any crimes in the future, whereas an individual belonging to 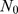 has not committed any crimes yet, but may in the future, if the occasion presents itself.

Upon committing crimes, players may or may not be arrested and punished. We assume that once a player has been arrested 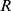 times, he or she is considered incorrigible and incarcerated until the end of the game, mimicking mandatory sentencing policies. Thus, after 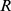 arrests players are taken out of the game and placed into the pool of unreformables 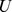. As a result, while players may transition between states 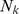, states 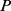 and 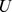 act as sinks with paladins and unreformables no longer involved in the game as active participants.

Finally, population conservation holds so that, at all times
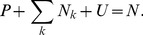
(1)


Note that players may have committed 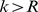 crimes before being arrested so that the summation over 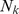 in Eq. 1 is in principle unbounded.

For simplicity, we will consider an initial population of players with no criminal history so that initial conditions are set as 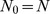, and 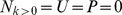. We follow societal dynamics from the neutral state 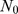 towards subsequent states 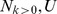 or 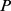 by assuming that when faced with the opportunity to commit a crime, players will decide to offend or not based on past history, apprehension likelihood, societal pressure, the threat of punishment but also, in case of recidivists, on possible forms of rehabilitation previously offered by society. As we shall later see, by construction, the game will end when all players are either paladins or unreformables, so that, eventually, 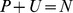. A quantity of interest throughout this work will thus be the 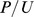 ratio, which we use as the final indicator of whether an ideal society is attained, with 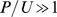, or whether instead a dysfunctional society emerges, with 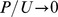. Note that in principle we could consider an open-ended game where criminals are continuously exposed to crime opportunities to which they respond depending on their past history. In this case, however, we would need to define a specific measure to describe the degree of optimality of a society, to replace the 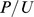 ratio. We choose to work with players irreversibly turning into paladins or unreformables since the 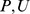 sinks naturally define 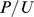 as a mathematically straightforward order parameter.

The game is played out in a succession of “rounds” 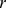. At each of these rounds, an individual 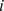 is selected at random from any of the 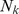 pools. We assume the individual in the group 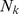 has a history of 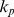 punished and 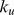 unpunished crimes, so that 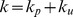. At each arrest and conviction the player is punished by an amount 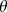 but also given educational and employment opportunities of magnitude 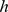 for a duration 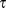. The dimensionless parameter 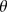 thus represents the stick of our game, while the parameters 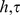 describe the carrot. Since decisions made by an individual depend on past criminal record, we describe each each player via a string containing punishment status and round of crime occurrence. We label each convicted crime by 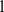 and each unpunished crime by 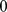. For example, if a player is in pool 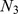 this implies there have been 3 crimes, committed at rounds 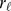 where 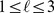. If we assume, say, that the first two crimes were left unpunished while the player was punished for the third one, the history string associated with individual 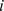 is 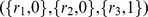. In this example 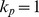 and 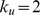.

Individual 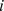 is now faced with the choice of whether to commit a new crime or not. We assume this occurs with probability 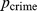 given by
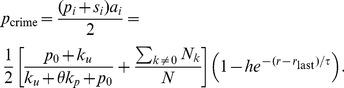
(2)


We choose this form – given by the sum of two terms, multiplied by an attenuating factor – to embody the assumption that individuals commit crimes depending on their own personal history[36], represented by 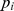, and on the surrounding community imprint[38], represented by 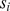, in equal manner. Given this crime propensity, we assume that the probability of committing a crime is finally modulated by the recidivism probability 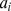, which includes any resources individual 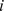 may have received in the past. In Eq. 2 we assume that if no crimes have been committed yet, 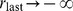 so that, effectively, no resources have been assigned either. Note that at the onset of the game when 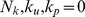, the overall probability to commit a crime is 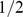, so that individuals are equally likely to offend or not.

We now examine the terms in Eq. 2 in more detail. The first term 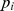 is the contribution to 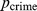 that strictly depends on the player's past history [36], given by
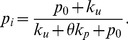
(3)


The form for the “stick” is chosen such that previous unpunished crimes 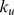 embolden the criminal, since 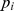 is an increasing function of 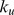. Similarly, previously punished crimes will hinder the likelihood of future offenses, since 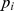 is decreasing in 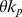. Note that 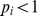 only when 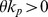: if 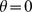 there are no consequences for committing crimes and players will always inherently want to offend, if 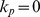 the criminal was never punished and feels emboldened by the impunity. The intrinsic crime probability 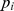 increases with 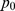 for all values of 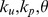. The parameter 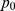 is also a measure of how sensitive 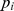 is to punishment after the first crime and apprehension. Consider the case 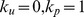. Upon differentiating 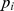 with respect to 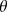 and setting 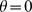 one finds
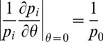
(4)so that larger values of 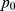 represent a smaller sensitivity to the 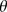 punishment. The next term in Eq. 2 is 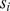, which represents a societal pressure term which we model by
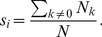
(5)


Including 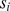 in 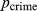 allows us to incorporate the assumption that crimes will generate more crimes, either by imitation, or by observed degradation of the community [38]. On the other hand, if the community is mostly comprised of virtuous 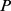 or neutral citizens 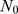, the societal pressure term is very small and so is the probability of committing crimes. In the limit of 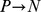, 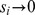.

Finally, the sum 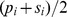 is attenuated by the factor 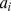 due to societal intervention evaluated at the last round player 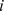 committed a crime. We model the effect of the “carrot” by the functional form
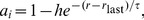
(6)where 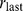 denotes the round number at which the last punished crime occurred. This term represents intervention and help from third parties, such as helping individual 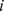 with employment, education opportunities, or, in the case of youth, the support of a mentor. We assume that these assistance programs are implemented with intensity 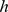 and decrease in time over a period 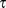. Note, from Eq. 6, that if 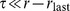 and rehabilitation programs are short lived, the exponent tends to zero, 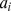 approaches 1, and there is no attenuation effect. On the other hand, if 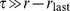, the attenuation is most effective at 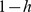. We assume 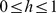. In principle, we could also let both 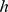 and 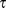 depend on crime number 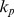, but for simplicity we will keep them constant for the remainder of this work.

After player 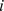 is faced with the opportunity to commit a crime, the game proceeds depending on the choices made. If the crime was not committed, the game proceeds to the strategy change phase; otherwise an apprehension and punishment phase play out. We assume that the apprehension and punishment probability is 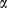, and that the odds of being arrested are known to criminals. We also assign resources 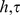 to a criminal every time he or she is arrested, regardless of their criminal past history. The final step of the game is for player 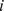 in population 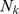 to update his or her strategy. We start with the possibility that the player has committed no crimes; in this case, he or she will either proceed to the paladin pool 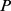 with probability
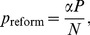
(7)or remain in the current subpopulation 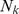 with probability 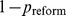. The underlying idea is that we assume player 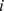 will commit to turning his or life around after having been “tempted” and not having caved in to crime. We further assume this decision depends on societal imprint expressed by the proportion of virtuous citizens, 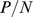 and modulated by 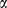, the probability of an arrest.

If player 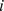 committed a crime but was not apprehended, he or she moves from pool 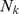 to pool 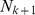 with probability 1. In this case, since there were no consequences for having committed crimes, we assume players likewise have no incentives not to commit criminal actions in the future. The last case is when a crime was committed, the criminal was apprehended and incentives for rehabilitation were assigned. Under this scenario, we assume that the criminal decides to turn into a law-abiding citizen and join the paladin pool 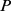 via the probability
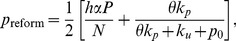
(8)while he or she will join the population 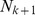 with probability 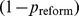. In Eq. 8 we assume that the reform probability depends both on societal imprint and on the player's punishment history. In particular, if no resources or punishment are offered and both 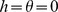 there is no incentive for players to reform. Note that if a player committed a crime during round 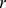, the 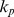 to be utilized when evaluating 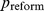 is the same at the onset of the round, augmented by one. For all parameter values 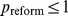.

Finally, we assume that when players are arrested 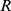 times they are considered incorrigible and are sentenced to lengthy incarceration periods that effectively take them out of the game and into the unreformable pool 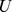. These players act only as bystanders and yield a negative imprint to society, just as paladins do but in a positive manner. By construction, our game will end when all players are either in subpopulation 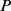 or 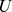. A majority of paladins represents a desirable,“utopian” society and a majority of unreformables an undesirable, “dystopian” one.

To summarize, the parameter space associated with our model consists of six quantities 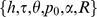. However, consistent with police estimates[39], we set the apprehension and punishment rate 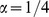 and we fix 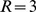 as the maximum number of punished crimes before players join the pool of unreformables 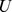. Thus, in the remainder of this work we only consider only the parameter set 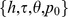. All parameters and variables of interest are summarized in Table 1.

**Table 1:** List of subpopulations and of relevant parameters.

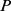	paladins
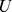	unreformables (who have have been punished 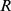 times)
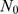	number of persons that have committed no crimes
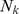	number of persons that have committed 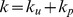 crimes
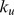	number of unpunished crimes per person
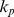	number of punished crimes per person
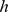	effective resource parameter
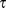	duration of assistance
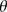	severity of punishment
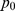	punishment sensitivity
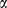	arrest and conviction probability
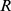	maximum number of punished crimes

We set 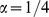 and 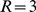 throughout this work.

## Methods

While statistical methods have been routinely used in the quantitative study of crime[40], [41], game theory approaches are a relatively new contribution. On the other hand, there is a quite rich literature on Monte Carlo methods for simulating games that involve decision making and strategy updating[42]. In this work, we implement our criminal game as a Monte Carlo simulation where we track the behavior of each individual over the duration of the game and where each round is a discrete time step. As mentioned in the previous section, a dynamic history string that summarizes past crime and arrest occurrences are assigned to each individual. For these, we evaluate transition probabilities between possible subpopulations 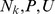 every time a decision process is involved.

At every round we select a random player within any of the 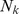 subpopulations and present him or her with the opportunity to commit a crime, evaluating 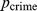 and 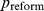 to inform decisions and strategy updates. We repeat this procedure for all 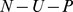 players and update the resulting 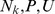 subpopulations only after the decision process has been carried out for all players, consistent with parallel update discrete time Monte Carlo methods[42]. We also calculate relevant crime, punishment and recidivism statistics at each round, until the end of the game, when all players are either in the 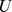 or 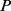 subpopulations. Finally, we generate contours of the ratio 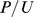 at the end of the game, which describes how ideal the outcome society is, given the parameters 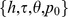.

Within our work, the average total number of crimes per player is evaluated as the sum of migrations between subpopulations 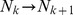 for 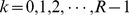 over the course of the game, normalized by the total number of players 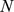. Similarly, the average total number of punishments per player is defined as the sum over increments of 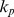 over the entire course of the game normalized by 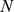. Finally, the average recidivist rate is the sum over increments of 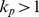 normalized by the total number of criminals who have been punished at least once[29]. In the Results section, we investigate how all of the above quantities vary with the model parameters 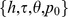 for a set of 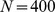 individuals. To limit the space defined by our four parameter model we limit 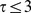 and consider only representative values of 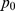, since – as we shall see – our results are monotonic in 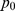. Parameters 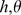 instead will be chosen as 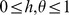, which are limitations imposed by the model.

For each criminal conviction, the justice system will impose an amount 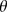 of punishment to the offender and an amount 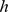, over an effective period 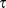, for rehabilitation and assistance, yielding a total rehabilitation cost of 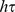. The latter is estimated by considering all resources on rehabilitation spent from the moment of arrest when 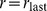 until the end of the game at 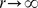, and using a continuum approximation so that
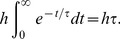
(9)


Since law enforcement may have limited total available resources 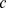 to both punish and rehabilitate a criminal we introduce the constraint 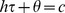. Higher punishment levels 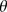 thus translate to lower rehabilitation efforts 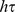, and *viceversa.* We will often invoke this constraint when examining the variation of derived quantities with respect to 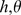.

## Results

In this Section we show and discuss results from our Monte Carlo simulations for different parameter choices. As discussed above, in analyzing our data we will use the resource constraint 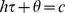. Note that the total number of crimes 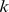 committed by a player can increase but not decrease, so that the dynamics is irreversible. We thus expect to find final configurations that depend on our specific initial conditions.

### Population dynamics

**Figure 1:**
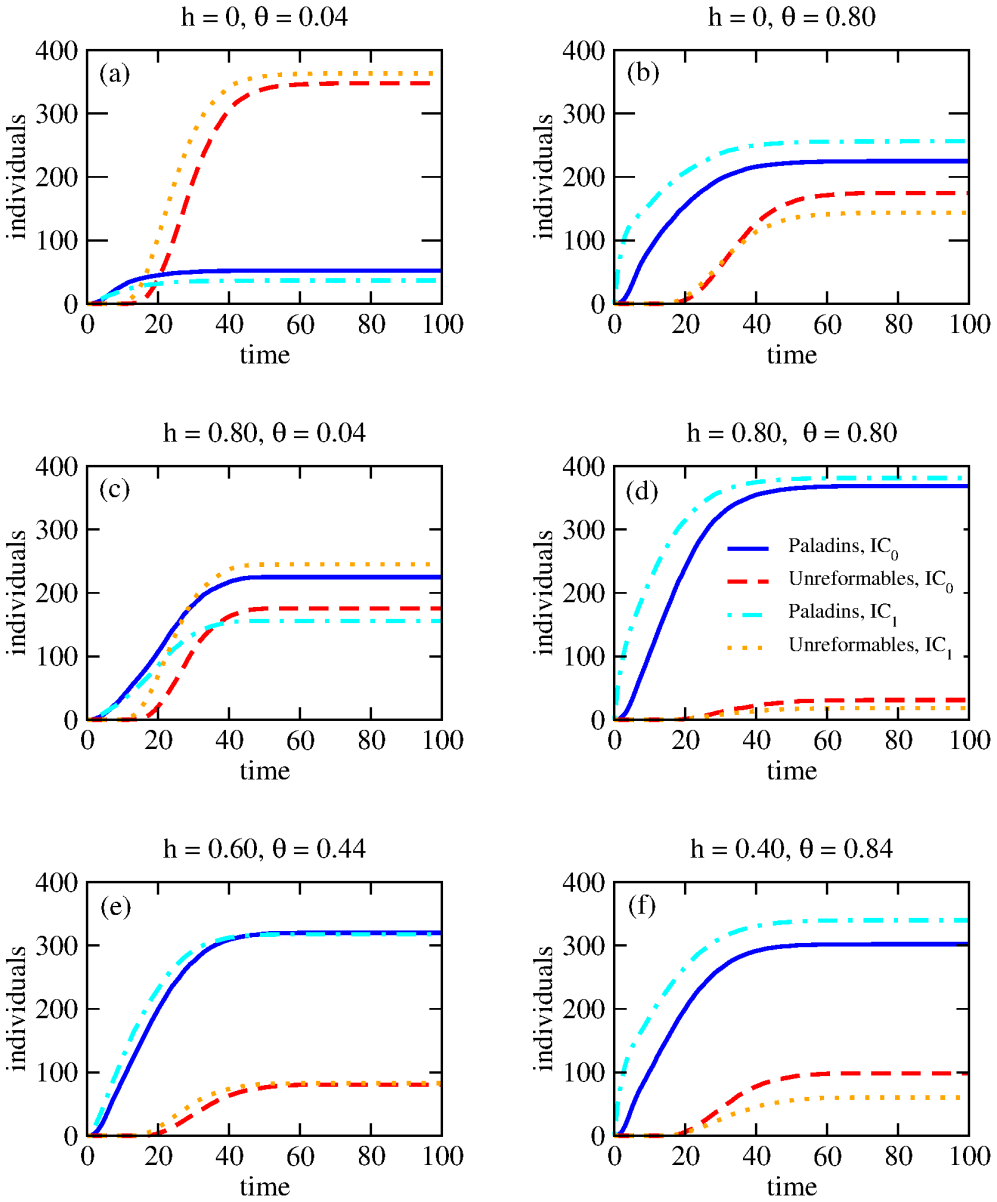
Dynamics of Paladins 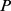 and Unreformables 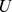 for 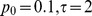 and variable 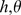 under initial conditions 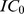 where 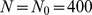 and 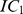 where 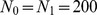. (a) For 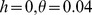, 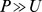, since punishment is low and no post-release resources are allocated. For 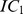 the number of unreformables is slightly higher than for 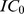. (b) For 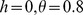, higher punishment leads to a deterrence effect and 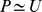. This trend is more evident for 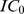 as explained in the text. (c) For 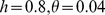, 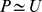. (d) For 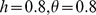 higher punishment leads to 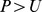. (e), (f) Dynamics under the constraint 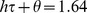 as in panel (c).

Since our game is constructed to evolve towards a final configuration where all players are either in subpopulation 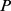 or 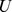, we follow the time evolution of the number of players in these states over the duration of the game. In Fig. 1 we show the dynamics of 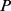 and 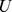 as the game progresses for various choices of 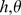 when 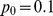 and 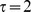. All curves are truncated at 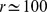, when 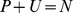 and the game ends. We use initial conditions 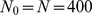 and 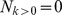 (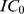) and 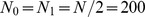 and 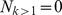 (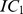) to investigate the effects of different starting choices. We let 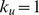 and 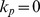 for all 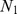 individuals within 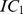 so that all players start the game without having been punished.

In Figs. 1(a) and (b) no resources are utilized for rehabilitation programs (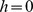). The punishment level is set to the low value 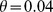 in panel (a), yielding a large number of unreformables for both sets of initial conditions, while for the higher punishment choice 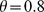 in panel (b) we find that the number of paladins exceeds that of unreformables 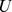. Note the slightly different behaviors for the two sets of initial conditions in panel (b): within 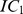 the initial society includes individuals with a criminal past at 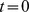, and the final number of paladins is greater than for initial conditions 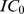 where all citizens started out in the neutral state. This difference arises because of the following. At the onset of the game 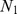 for 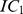 is greater than 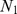 for 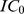; due to the structure of 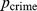, more crimes will be initially committed for 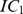 than for 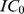. The high value of 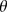 in 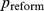 will lead players who are arrested to more likely reform, increasing the number of paladins and decreasing 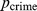. This leads to a feedback loop that effectively keeps increasing the number of paladins throughout the game and that is larger for 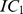 than for 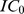 due to the initial conditions.

In Figs. 1(c) and (d) we keep the punishment levels equal to those used in panels (a) and (b) respectively but include the assignment of resources 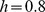 over a time 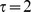. As shown in Figs. 1(c) and (d) adding resources dramatically increases the final number of paladins. The behavior in panel (c), where there are a large amount of resources but little punishment, is interesting: within 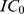 the number of paladins at the end of the game is greater than that of unreformables, but within 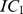 the opposite holds, showing the importance of initial condition choices. In particular, within 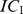, the initial presence of a large cohort of players with a criminal past leads to a feedback loop where more crimes are encouraged since punishment is low, leading to a large 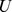 population. This effect is less pronounced within 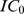 where players all start in the neutral state.

In Figs. 1(e) and (f) we keep the same total amount of resources as in Fig. 1(c), 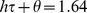, but use a different realization of the constraint: in panel (e) we allow for fewer resources 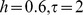 and more punishment 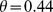 while in panel (f) we decrease the amount of resources even more, with 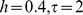 and 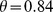. Given the 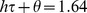 constraint, a comparison of panels (c), (e) and (f) shows that the relative number of paladins with respect to unreformables can be maximized by optimally modulating the parameter subset 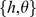. In particular for 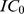, out of the three panels (c), (e), (f) examined, the parameter choice in (e), with the optimal balance of punishment and rehabilitation efforts, is the most effective in yielding the largest final 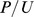 ratio. On the other hand, for 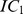, panel (f) yields optimal results. We will later explore parameter space more in detail and study the final 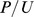 ratio over a wider range of 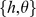 values.

Finally, in all panels of Fig. 1, we observe a slight delay in the increase in 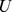 compared to the initial dynamics of 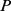. This is because player reform may occur from the beginning of the game, while for an individual to join the 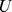 subpopulation he or she must have committed at least 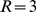 crimes.

### Correlations between 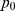 and 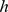


**Figure 2:**
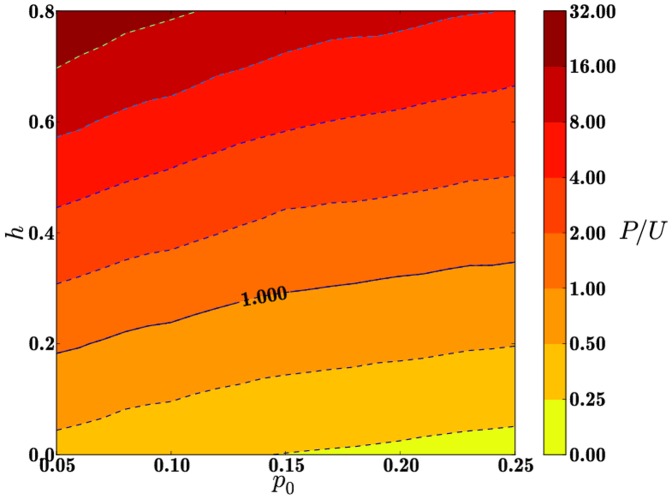
Contours of the final 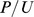 ratio as a function of 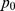 and 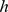 for 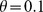, and 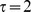 for 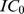. Note that the final 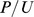 ratio is an increasing function of 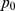 and 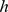. The solid curve marks the locus 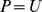.

In this subsection we investigate the role of 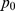 on the final value of the 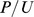 ratio. Since 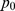 appears only in Eq. 2, and 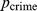 is an increasing function of 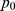, we expect all results to be similarly increasing in this parameter. In Fig. 2, we plot contours of the final 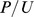 ratio as a function of 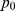 and 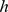 for 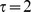 and 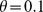 using initial conditions 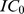. As expected, the final 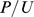 ratio increases both in 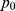 and 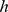. In Fig. 2 we have also highlighted the 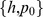 curve where the ratio 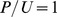. Note that for higher values of 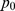, where 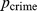 is higher, more incentives for rehabilitation 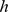 are needed to yield a final society comprised of equal numbers of paladins and unreformables. In this case, introducing the total resource constraint 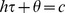 is equivalent to selecting slices in Fig. 2 at fixed 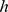. The resulting trend is clear: for fixed 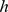 better results are obtained on a low 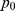 population, where the intrinsic probability 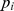 to commit crimes is lower. All other quantities of interest yield similar monotonic trends – namely, the crime, punishment and recidivism rates are decreasing functions of 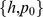 and we do not show them here. Similar considerations apply to initial conditions 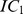.

### Correlations between 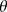 and 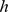


**Figure 3:**
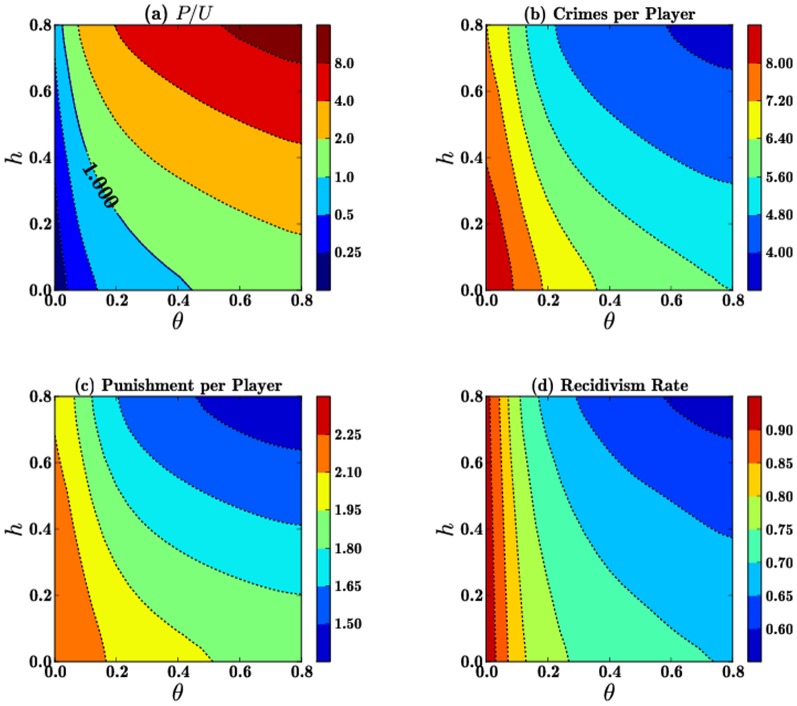
Contours of the final values of (a) the 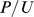 ratio, (b) the number of crimes per player, (c) the number of punishments per player and (d) the recidivism rate as a function of 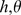 for 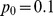 and 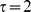. Initial conditions 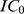 are chosen so that at the onset of the game 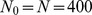, and 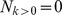.

In this subsection we study how all quantities of interest vary within the 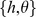 parameter space for initial conditions 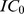, and for 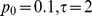. Qualitatively similar results arise for different values 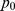, so we keep this parameter fixed. In Fig. 3(a) we show that the final 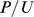 ratio increases with both 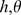 while the total number of crimes and punishments and the recidivism rates in Figs. 3(b),(c) and (d), respectively, decrease with 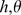. These are predictable trends since increases in both rehabilitation and punishment tend to drive overall crime down. In particular, note that punishment per player values in panel (c) are approximately one–fourth of the crimes per player, shown in panel (b). This is expected, since the punishment probability is given by 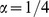.

**Figure 4:**
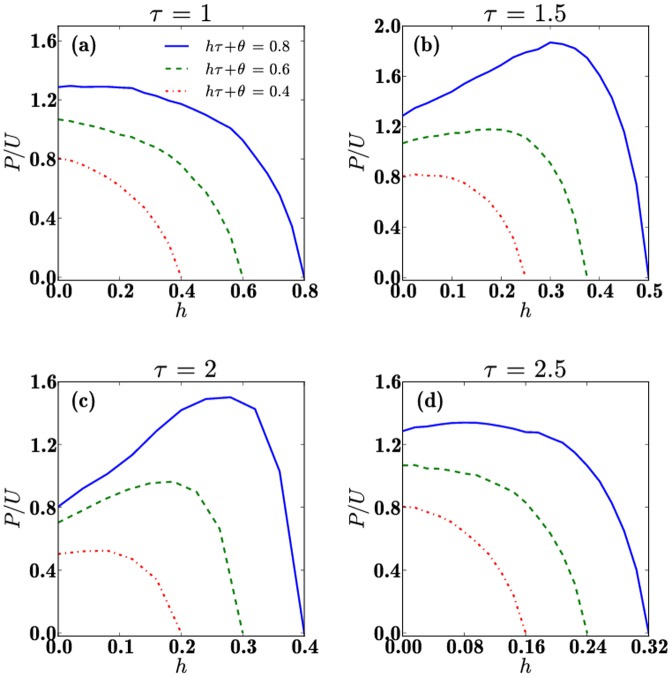
The final 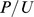 ratio plotted as a function of 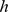 under the constraint 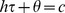, for (a) 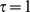 (b) 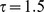 (c) 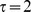 and (d) 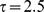. The constant is chosen as 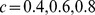 so that three curves are shown for each each value of 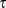. Each curve terminates at 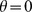. Panel (b) is projected from Fig. 3(a). Note that for all 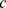 values, the most efficient allocation of resources is attained for the intermediate 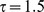. In particular, for 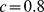 the final 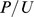 ratio is attained at 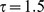, 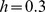 and 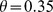 as shown in panel (b). Also note the emergence of maxima in panels (b) and (c).

We now introduce the constraint 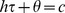. In particular, in Fig. 4(b), we show the final 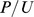 ratio as a function of 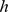 on the locus 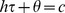 for 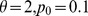 to mirror the parameter choices made in Fig. 3. The three curves are for the constant set at 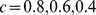, so that higher constants yield higher 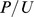 values at the end of the game. The most interesting feature we observe is that optimal values of 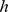 and 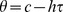 exist that yield maxima in the final 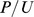 ratio. This implies, as mentioned earlier, that if law enforcement agencies have limited resources at their disposal to both punish and rehabilitate criminals, a proper balancing of these efforts may yield the best outcome in crime abatement. Furthermore, note that for small values of 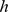, when 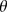 is high, increasing the levels of rehabilitation 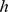 is beneficial, but that beyond a certain threshold, when 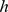 is too large and little punishment 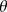 is assigned to criminals, the final ratio 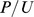 starts decreasing, implying that both punishment and rehabilitation are necessary. While a similar behavior is found in Fig. 4(c) for 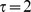 different trends are observed in Figs. 4(a) and (d) where 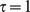 and 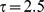 respectively. In the latter cases, the final ratio 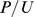 does not change appreciably as 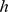 increases for low 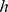. We notice instead a quasi-plateau regime, where increasing 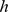 and decreasing 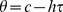 does not significantly affect the final 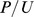 ratio. However, increasing 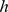 and decreasing 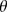 further leads to decreases in the final 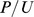 ratio: just as in Figs. 4(b) and (c) a threshold punishment level 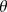 is necessary to keep 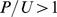 at the end of the game. Overall, the largest 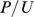 ratio is attained for 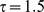, 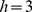 and 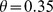, when the number of paladins is double that of unreformables.

Within the context of our model we find that if rehabilitation efforts are either too short or too long-lived they may be ineffective: in the first case because they do not last long enough to affect the criminal decision process, in the second case because long intervention programs with finite resources necessarily imply that these programs are not impactful enough and will have marginal effects on crime rates. Our findings imply that the best approach to minimize the final 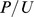 ratio is to punish the criminal adequately while leaving enough resources to be used over an intermediate period of time towards the criminal's rehabilitation.

**Figure 5:**
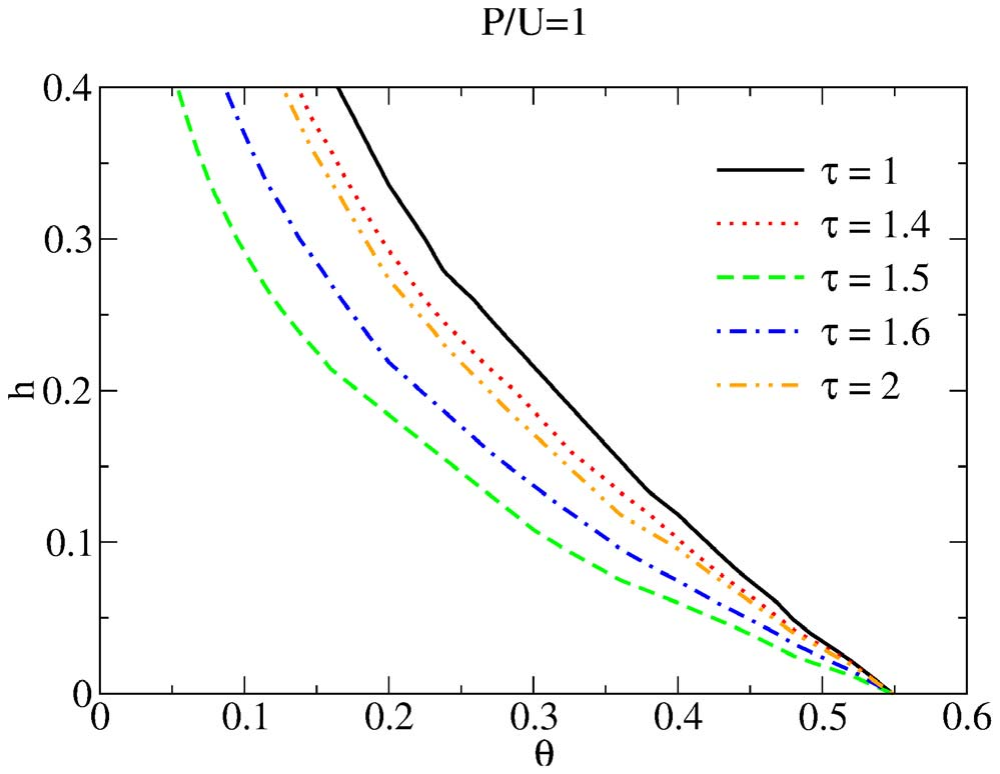
Curves along which at the end of the game 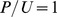 for different values of 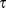. Given 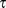, the area to the right of each curve corresponds to values of 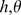 where 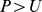 and the area to the left of each curve corresponds to values of 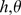 where 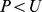. The curve for 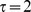 is projected from Fig. 3(a). When no rehabilitation resources are assigned (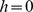) 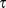 does not play a role so curves intersect at the same value of 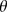. Note that the 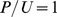 curve is lowest for 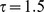, implying that for given 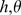 the best way to populate society with an equal amount of paladins and unreformables is by selecting an intermediate value for 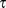. As explained in the text, intervention programs that are too brief or too long long yield less efficient results.

This trend is confirmed in Fig. 5, where we plot contours corresponding to 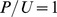 at the end of the game in 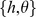 space for various values of 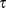 and for 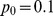. Note that rehabilitation programs lasting for intermediate times 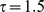 yield the lowest lying curve, indicating that equal numbers of paladins and unreformables can be attained for lower resource 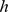 and punishment 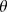 if intervention programs are neither too stretched out in time, nor too short.

**Figure 6:**
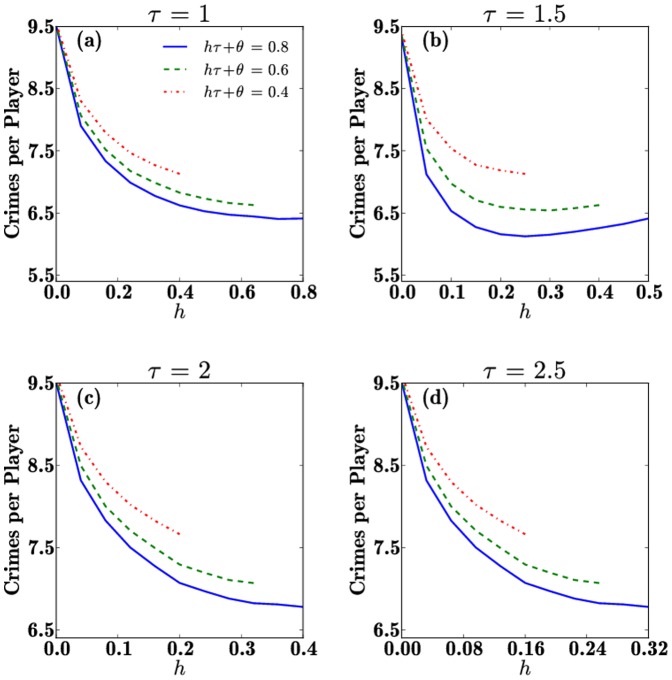
The number of crimes per player over the course of a game plotted as a function of 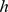 under the constraint 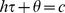, for (a) 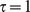 (b) 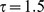 (c) 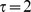 and (d) 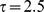. The constant is chosen as 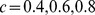 so that three curves are shown for each each value of 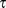. Each curve terminates at 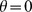. Note that a minimum arises in the case of 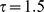 indicating that an optimal allocation of rehabilitation and punishment resources exists to minimize crime occurrences.

**Figure 7:**
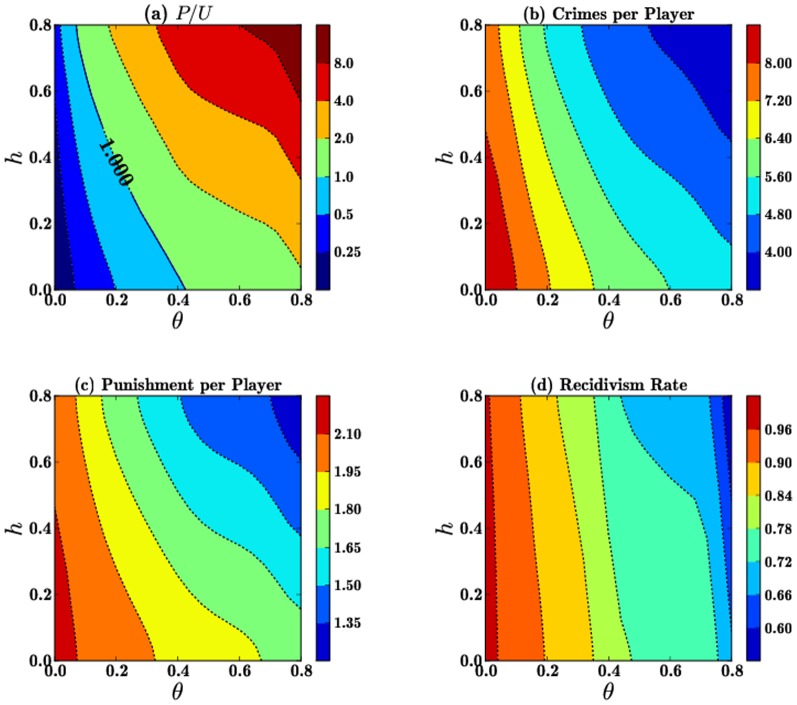
Contours of the final values of (a) the 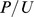 ratio, (b) the number of crimes per player, (c) the number of punishments per player and (d) the recidivism rate as a function of 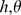 for 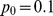 and 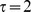. Initial conditions 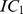 are chosen so that at the onset of the game 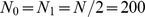, 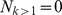 and all players within 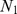 are assigned 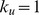 and 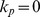. Note that while qualitative trends mirror the results shown in Fig. 3 for 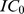, there are quantitative differences between the two different initial conditions.

In Fig. 6 we plot the number of crimes per player throughout the game as a function of 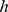 for the same parameters, 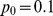, 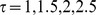 and the same constraints used in Fig. 4. As can be seen from panels (b) and (c) a minimum in the crime rates may arise depending on parameter choices, partly mirroring the results found in Fig. 4. Note that for 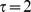 there is no minimum in the crime per player curves, which instead arises within the 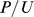 plots. Similar trends may be found for the total number of punishments per player and for the recidivism rates. Together with our findings for the final 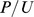 ratio, these results show that the occurrence of crime can be mitigated by properly balancing the partitioning of resources between punishment and rehabilitation. Finally, in Fig. 7 we show the equivalent results of Fig. 3 for the case of 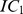. Note that although quantitatively different, the main features are similar from those obtained using 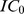.

### ODE-s corresponding to the model

In order to obtain a qualitative description of the model, we formulate the dynamics in terms of ordinary differential equations (ODEs) for the relevant subpopulations. These “mass-action” type ODEs implicitly correspond to random sequential updating and are not expected to match exactly our simulation results, obtained using parallel update dynamics. Nonetheless, we expect such an approach to yield qualitatively valid results, with significantly less computational effort. Due to the complexity of the game and to history–dependence events, the dynamics cannot be reduced to a set of equations describing the time evolution of 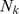, the number of players that have committed 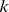 crimes. Instead, we must keep track of how many crimes were punished and how many were not, leading to an expanded population. We thus introduce 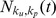 as the number of individuals who have committed 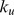 unpunished crimes and 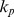 punished ones until time 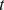 and study its evolution towards states with increasing 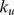 or 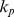 or towards the two possible sinks, 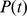 or 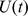. We choose to measure time in units of a single simulation update so that all probabilities used in our simulation rounds may be recast as rates per unit time. For notational simplicity we set 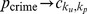, 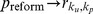. The mass-action rate equations can be expressed as
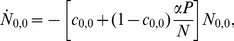
(10)

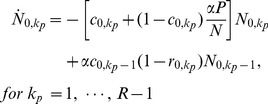
(11)

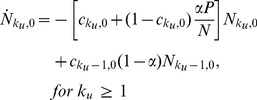
(12)

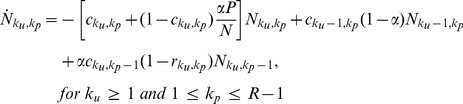
(13)


For 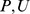 we write
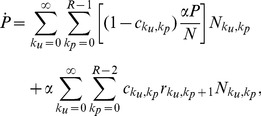
(14)

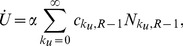
(15)where the 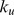 index and summations are unbounded. In the above equations, 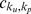 and 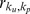 are derived directly from Eqs. 2 and 8 respectively
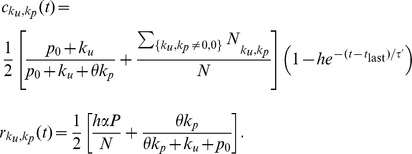
(16)


It can be easily verified that population conservation holds, since
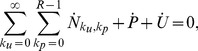
(17)for all times. Note that the dynamics contained in Eqs. 10–16 are irreversible. If we take the 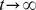 limit in Eqs. 10–16, we find 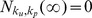 for all 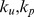 and 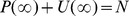, but no independent constraint on 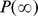 or 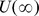. The ratio 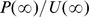 therefore, needs to be determined from the evolution of the dynamics and the specific initial conditions.

In order to numerically integrate Eqs. 10–16 we must first approximate 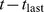 in Eq. 16. Note that for players committing their 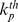 crime at time 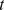, there is a 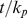 interval between arrests, so that we can reasonably assume 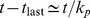. As in our numerical simulations, if 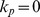, 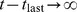, and there is no attenuation effect since no resources have been assigned to players who have never been punished. Since we are deriving continuous ODE-s starting from parallel update Monte Carlo simulations, an effective 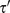 in Eq. 16 is required, which we estimate to be of the order of 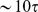. The rescaled 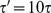 will largely compensate the difference between our parallel update simulations and the sequential update in the ODEs. Finally note that Eqs. 10–16 form an infinite set because 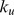 may grow indefinitely. Thus, in order to numerically implement our ODE system, an appropriate truncation scheme is necessary. We assume that for large enough 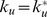, players join the pool of ‘uncatchable’ criminals, truncating the 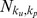 hierarchy at 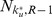. Our effective system is now made of 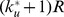 coupled equations in addition to the two sink equations for 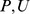 and a closure equation for 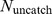 that can be written as follows
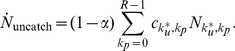
(18)


**Figure 8:**
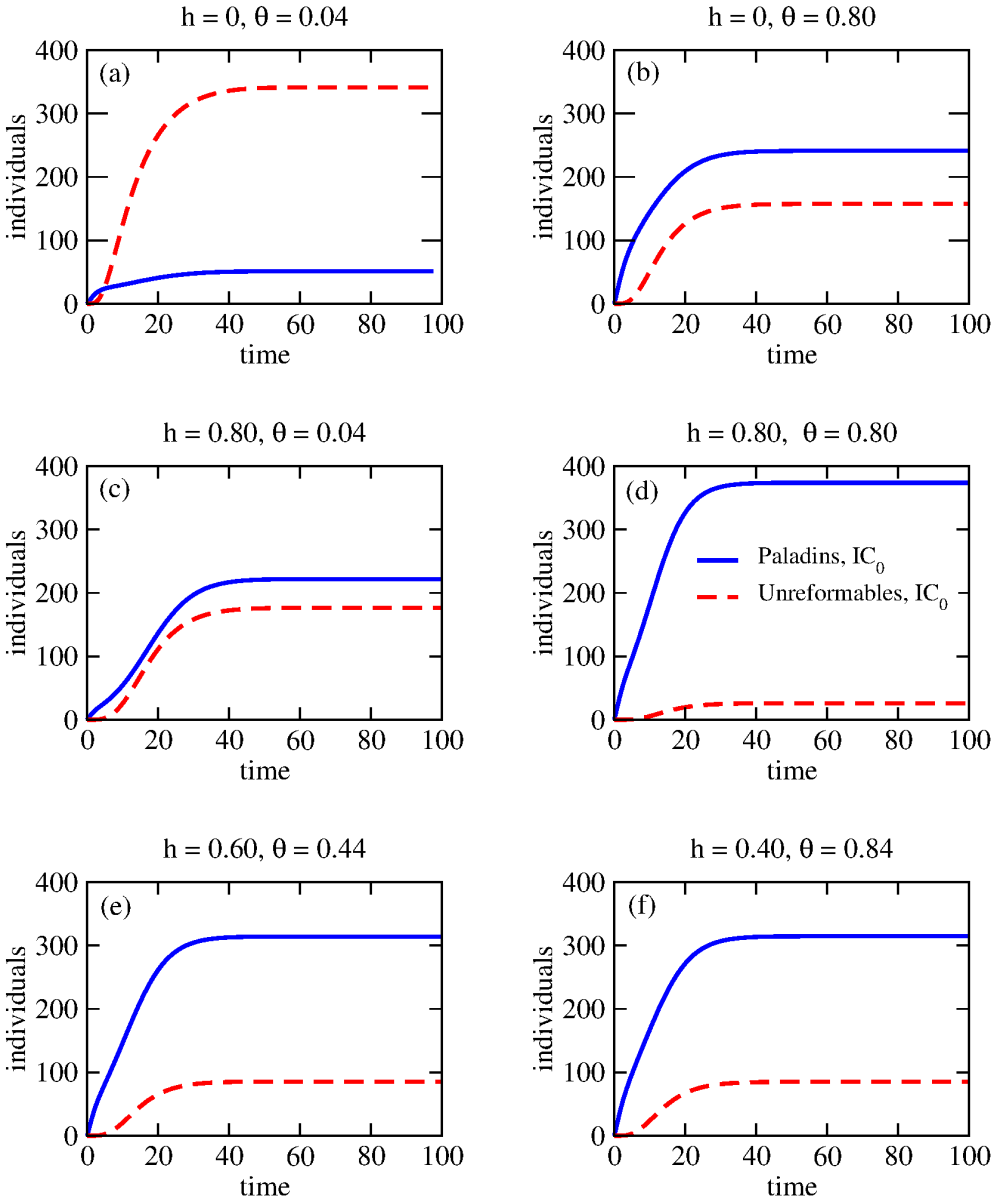
Dynamics of Paladins 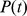 and Unreformables 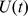 according to the ODEs in Eqs. 10-16 for 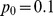, 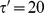 and variable 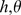 under initial conditions 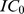 where 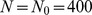. Note that the dynamics are qualitatively similar to the simulation results shown in Fig. 1.

The truncation scheme described above should not lead to large discrepancies with our simulations if 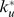 is sufficiently large, since the likelihood of being neither arrested nor of reforming – and thus ending in either the 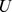 or 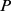 sinks – is small. We set 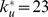 and verified in all cases that only a handful of players are able to reach the “uncatchable” status. We also verified that slightly smaller choices of 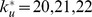 essentially lead to the same results. In Fig. 8 we plot the dynamics obtained from our set of coupled ODEs under the 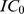 initial conditions, when 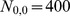 and 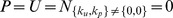. As can be seen, the agreement with our simulation results in Fig. 1 is very good. A similar qualitative agreement holds for 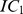, where 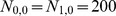 and 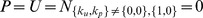 and which we do not show here.

## Summary and Discussion

We have proposed an evolutionary game that incorporates both punishment – the “stick” – and assistance – the “carrot” – to study the effects of punishment and rehabilitation on crime within a model society of 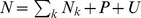 individuals. At every round, each of the 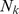 players that have committed 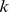 crimes may reoffend, and join the 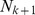 pool, or choose not to reoffend and remain in the 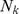 pool. We also allow players within 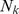 that choose not to reoffend to join the paladin pool 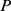 of players that will not commit any more crimes in the future. Finally, upon being arrested 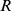 times, players join the pool of unreformables 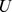. Within this context, the index 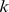 also represents how hardened or experienced the criminal has become.

Our model was studied via Monte Carlo simulations and via an approximate system of ODEs. From both approaches we find that increasing the severity of punishment as well as the magnitude and time duration of intervention programs yield lower incidents of crime and recidivism rates. Since in realistic scenarios total resources available to law enforcement may be finite, we also include a constraint 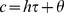 on the total punishment 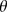 – the stick of our game – and on the rehabilitation resources 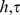 – the carrot of our game – so that increasing one effort will necessarily decrease the other. We find that an optimal allocation of resources may exist to minimize recidivism and crime rates, reinforcing the emerging viewpoint that a mixture of sufficient punishment and long–lasting assistance efforts upon release may be the most effective way to reduce crime.

From a mathematical point of view, the continuum ODEs we derived correspond to random sequential updating processes, rather than to the parallel updating schemes used in our simulations. We have shown that by considering rescaled time scales, and for some parameter regimes, results from the ODEs we derived are qualitatively similar to the simulated ones. However it would be mathematically interesting to derive the corresponding continuum equations directly from our parallel updated simulations and compare how they differ from the current ODEs.

Several “carrot and stick” evolutionary games and experimental studies have been presented in the literature, especially in the context of public goods games [14], [23], [43], [44]. In most cases, cooperators are rewarded with incentives and defectors punished, and in some instances players have the extra option of non-participating [14]. A common finding is that, to varying degrees, incentives promote cooperation [22], [45], [46], with punishments further enhancing the level of cooperation among players [15]. Our work differs from the above scenarios in that instead of assigning punishments or rewards to players depending on their cooperative or defective behaviors, we both punish and rehabilitate defectors, so that their carrot and stick experiences are not mutually exclusive and that any player's future behavior depends both on how much each he or she was punished and on the quality and duration of incentives for rehabilitation he or she has received. Although the way we assign incentives and punishments differs from standard “carrot and stick” games, our results confirm that punishment and rewards complement each other and that both tools should be used by law enforcement to reduce recidivism.

Within our work, rehabilitation resources were specified via the collective parameter 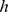. However, various rehabilitation opportunities are possible – in the form of educational or vocational training, behavioral treatments, or fostering family relationships. Each of these comes with possible modeling opportunities and challenges that are beyond the scope of this work. We have also made numerous assumptions in our work by neglecting effects of heterogeneity in age, race, gender or other socio-economic or geographical considerations on 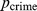 and 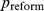. We have assumed all-to-all couplings between players so that each individual's choices depend on the entire society. The introduction of a dynamical network where each individual is linked to friends, family and employers that selectively influence each player's decisions, could represent a more realistic approach. Finally, we have kept the arrest probability 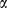 fixed and assumed that rehabilitation efforts were assigned to all players, with a fixed magnitude and time duration, regardless of the player's history and have not included incarceration periods between crime events. Including all these refinements would add more complexity to the underlying model; whether and how they may change our results will be the subject of future investigation.
